# Molecular dynamics simulation reveals how phosphorylation of tyrosine 26 of phosphoglycerate mutase 1 upregulates glycolysis and promotes tumor growth

**DOI:** 10.18632/oncotarget.14517

**Published:** 2017-01-05

**Authors:** Yan Wang, Wen-Sheng Cai, Luonan Chen, Guanyu Wang

**Affiliations:** ^1^ Department of Biology, Southern University of Science and Technology, Shenzhen 518055, China; ^2^ Research Center for Analytical Sciences, College of Chemistry, Nankai University, Tianjin 300071, China; ^3^ Key Laboratory of Systems Biology, CAS Center for Excellence in Molecular Cell Science, Innovation Center for Cell Signaling Network, Shanghai Institute of Biochemistry and Cell Biology, Chinese Academy of Sciences, University of Chinese Academy of Sciences, Shanghai 200031, China

**Keywords:** oncotarget, glycolysis, molecular dynamics simulation

## Abstract

Phosphoglycerate mutase 1 (PGAM1) catalyzes the eighth step of glycolysis and is often found upregulated in cancer cells. To test the hypothesis that the phosphorylation of tyrosine 26 residue of PGAM1 greatly enhances its activity, we performed both conventional and steered molecular dynamics simulations on the binding and unbinding of PGAM1 to its substrates, with tyrosine 26 either phosphorylated or not. We analyzed the simulated data in terms of structural stability, hydrogen bond formation, binding free energy, etc. We found that tyrosine 26 phosphorylation enhances the binding of PGAM1 to its substrates through generating electrostatic environment and structural features that are advantageous to the binding. Our results may provide valuable insights into computer-aided design of drugs that specifically target cancer cells with PGAM1 tyrosine 26 phosphorylated.

## INTRODUCTION

Glycolysis is a pathway that converts one molecule of glucose to two molecules of pyruvate, with the concomitant generation of two molecules of adenosine triphosphate (ATP). The latter is known as the energy currency for the cell, because it provides energy for virtually any biological processes such as biosynthesis of macromolecules. Glycolysis is of critical importance, because it bases all the other pathways of glucose metabolism; and unlike the other pathways, it requires no participation of oxygen, thus conferring survival advantages for those rapidly dividing cells such as cancer cells, which are often in the hypoxic state. Even when oxygen is abundant, cancer cells tend to avoid oxygen dependent glucose metabolism by upregulating glycolysis, a phenomenon long known as the Warburg effect [1]. The Warburg effect implies that glycolysis may possess some advantages [1, 2]. Therefore, studying glycolysis may provide valuable insights into our understanding of cancer metabolism. Glycolysis consists of ten steps.

**Figure 1:**
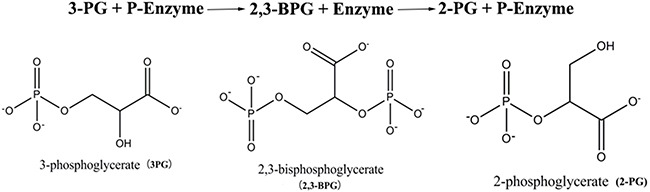
The phosphoglycerate mutase reaction and the chemical structural formula of 3PG, 2,3-BPG, and 2PG

Phosphoglycerate mutase 1 (PGAM1) is an enzyme catalyzing the eighth step of glycolysis, namely isomerization of the substrate 3-phosphoglycerate (3PG) into 2-phosphoglycerate (2PG) (Figure 1). 3PG and 2PG differ only in the location of the phosphoryl group, which is at position C-3 in 3PG and C-2 in 2PG. The isomerization starts with an active PGAM1, namely PGAM1 with its histidine 11 residue (H11) phosphorylated. The isomerization can be divided into two half reactions [3, 4]. First, PGAM1 binds with the substrate 3PG and transfers its phosphoryl group to 3PG at position C-2, generating the intermediate 2,3-bisphosphoglycerate (2,3-BPG). Second, the phosphoryl group at C-3 of 2,3-BPG is transferred to H11 of PGAM1, whereby 2,3-BPG turns into the product 2PG. The phosphorylation of H11 reactivates PGAM1, which can catalyze a new round of isomerization.

Upregulation of PGAM1 is implicated in the development of many cancers, including hepatocellular carcinoma and colorectal cancer [5, 6]. Rapid growth and division of cancer cells depend critically on their capability of coordinating glycolysis and biosynthesis, in which PGAM1 plays a crucial role. Hitosugi et al. found that PGAM1 contributes to the coordination by controlling intracellular levels of its substrate 3PG and product 2PG [7]. There exist two mechanisms to explain PGAM1 upregulation. First, the loss of *TP53* leads to increased expression of *PGAM1*, which is a negative transcriptional target of *TP53* [8–10]. The second putative mechanism is the phosphorylation of tyrosine 26 (Y26) residue of PGAM1, which may greatly enhance PGAM1 activity and can explain the finding that Y26 phosphorylation is commonly found in human cancers [2]. To understand how Y26 phosphorylation may enhance PGAM1 activity, Hitosugi et al. crystalized human PGAM1 proteins with both phosphorylated and dephosphorylated H11 [2]. Comparison of the two structures suggested that Y26 phosphorylation causes a conformational change that is characterized by the leaving of the negatively charged glutamic acid 19 (E19) residue from the active site, thus promoting 2,3-BPG binding and consequently H11 phosphorylation. This may also help to keep the active site open for substrate (3PG) binding. In brief, the active site of PGAM1 may be partially blocked by E19 when Y26 is not phosphorylated; and Y26 phosphorylation may clear the blockage and thus enhance PGAM1 activity. Although reasonable, the explanation was based on static data and lacked dynamical evidences, including computer simulations of atomic movements whereby the enzyme-substrate binding is achieved. Indeed, the crystal structures were only two snapshots containing no such dynamical information.

In the present paper, we used molecular dynamics (MD) simulation to test the hypothesis that Y26-phosphorylation enhances the activity of PGAM1 and to learn how the enhancement is achieved. MD simulation is a computational method for studying the physical movements of atoms and molecules, which are allowed to interact for a fixed period of time, giving a dynamical evolution of the system [11–14]. It is very useful in exploring enzyme-substrate interactions [15].

We first built *in silico* the complex 2,3-BPG:PGAM1, formed by binding of 2,3-BPG to PGAM1. The complex is called the wild type system or the Y26-phospho system, when Y26 is dephosphorylated or phosphorylated, respectively. In the following, we also use a suffix *wt* or *phos* to signify Y26's phosphorylation state. For example, PGAM1 *phos* represents PGAM1 with Y26 phosphorylated. We then performed MD simulations on both systems to obtain the detailed atomic movements that facilitate the binding of 2,3-BPG to PGAM1 and then calculated the binding free energy. We also studied the binding of PGAM1 to 3PG and 2PG, respectively. By comparing the results, we explained how Y26 phosphorylation enhances PGAM1 activity and glycolysis.

These results may provide further insights into tumor growth and lead to drug targets for cancer by e.g. preventing Y26 phosphorylation.

## RESULTS & DISCUSSION

### General structural features of 2,3-BPG:PGAM1

**Figure 2:**
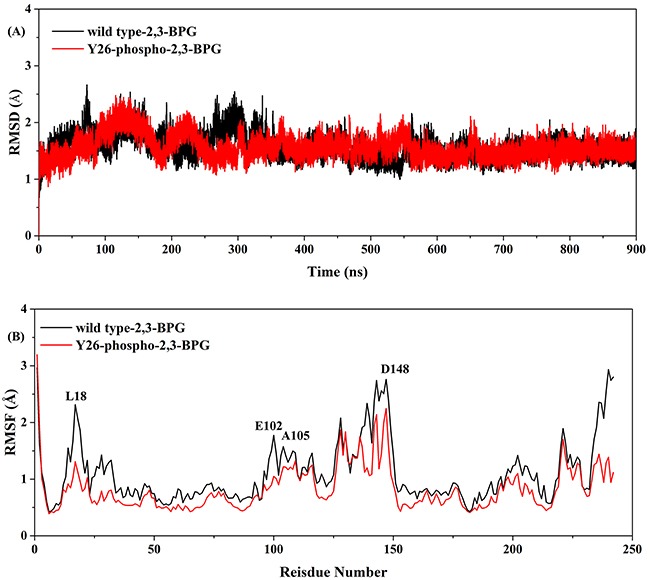
**A**. The time course of RMSD values of the wild type system (black) and the Y26-phospho system (red). **B**. The time course of RMSF values of the two systems. Those residues with high RMSF values are labeled.

For each of the two systems, 900 ns MD simulation was carried out, which generated 450000 frames of trajectory data. To assess the system's structural stability, we calculated RMSD of C-α atoms of the protein backbone, by using the software AmberTools15. As shown in Figure 2A, both systems underwent fierce conformational changes during the first 300 ns, with the wild type system changing greater. Notably, the RMSD values of the wild type system exceeded 2.5Å several times, while those of the Y26-phospho system were all below 2.5Å. From 300 ns to 700 ns, the RMSD values of both systems fluctuated around 2.0Å, with the wild type system having a slightly larger magnitude. Fluctuations reduced greatly during the last 200 ns, with the average RMSD value reaching ~1.5Å in both systems. The wild type system still fluctuated slightly more intensive than the Y26-phospho system. In line with the RMSD results, RMSF analysis also demonstrated the greater conformational variations of the wild type system (Figure 2B), indicating that residues of the wild type system are generally more flexible than those of the Y26-phospho system. The flexibility difference was most dramatic at residues spanning different regions of PGAM1: Leucine 18 (L18), Glutamic acid 102 (E102), Alanine 105 (A105), and Aspartic acid 148 (D148). Therefore, both RMSD and RMSF analyses suggested that Y26 phosphorylation stabilizes the binding of 2,3-BPG.

The scenario may be different during periods far away from stabilization of binding. During the entry into or exit from the binding site, the enzyme may undergo significant conformational changes; and events such as mutation and single residue phosphorylation (the present case) increase the likelihood of their occurrence [16–26]. To observe such conformational changes, one has to wait a long time until the ligand egresses from the binding site, if the conventional Molecular Dynamics (cMD) simulation is used. We thus used Adaptive Steered Molecular Dynamics (ASMD) to accelerate the simulation, with the RMSF results presented in Supplementary Figure S4 (red) against the original cMD RMSF data (black). For the wild type system, ASMD and cMD yielded close RMSF results. The most dramatic changes occurred at residues 125 to 140, which were not significantly involved in binding. For the Y26-phospho system, RMSF values increased markedly of residues K113 to R117, which were all prominent binding residues. These data imply that Y26 phosphorylation may facilitate the initial binding of 2,3-BPG to PGAM1 by making the binding pocket open wider, namely by inducing greater conformational changes of the binding pocket.

The flexibility difference can be explained by other techniques of trajectory analysis, such as the following hydrogen bond analysis.

### Hydrogen bond formation in the Y26-phospho system stabilized 2,3-BPG binding

**Figure 3:**
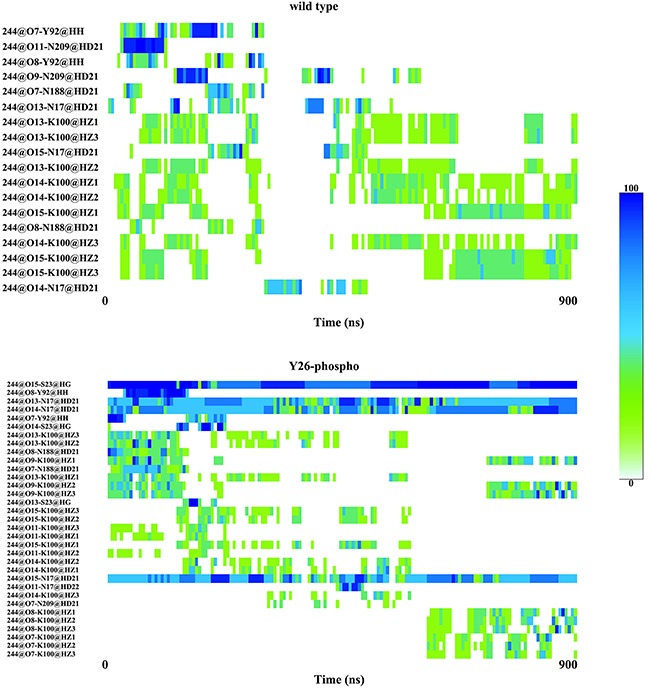
Frequency of hydrogen bond formation between 2,3-BPG and its adjacent PGAM1 residues, for both the wild type system (upper panel) and the Y26-phospho system (bottom panel) The rows correspond to various hydrogen bonds formed. The columns correspond to time zones.

Non-bonded interactions such as hydrogen bond, hydrophobic interaction, salt bridge play important roles in macromolecules’ structural formation and biological functions. We examined the formation of hydrogen bonds between 2,3-BPG and its surrounding residues of PGAM1 over the 900 ns simulation for both the wild type system and the Y26-phospho system, with the results presented in Table 1 and Figure 3. Hydrogen bond formation was based on the two criteria presented in the Method section. We found that the wild type and Y26-phospho systems had 18 and 33 hydrogen bonds formed, respectively. That is, 2,3-BPG molecule in the Y26-phospho system formed more hydrogen bonds than in the wild type system. More importantly, the hydrogen bonds persisted much longer in the Y26-phospho system than in the wild type system, indicating that PGAM1 *phos* interacts more actively with 2,3-BPG, than PGAM1 *wt* interacts with 2,3-BPG.

**Table 1:** Properties of the formed hydrogen bonds between 2,3-BPG and its adjacent PGAM1 residues

(a) the wild type system				
#	H-bond	Occupancy (%)	Distance (Å)	Angle (°)
1	244@O7-Y92@HH	10.82	2.68	161.78
2	244@O11-N209@HD21	8.51	2.79	164.14
3	244@O8-Y92@HH	6.26	2.66	159.88
4	244@O9-N209@HD21	10.24	2.78	161.64
5	244@O7-N188@HD21	5.41	2.83	157.35
6	244@O13-N17@HD21	9.50	2.78	155.22
7	244@O13-K100@HZ1	8.17	2.80	151.06
8	244@O13-K100@HZ3	7.92	2.79	151.84
9	244@O15-N17@HD21	6.44	2.81	157.34
10	244@O13-K100@HZ2	7.32	2.79	150.19
11	244@O14-K100@HZ1	9.09	2.79	149.13
12	244@O14-K100@HZ2	8.10	2.79	149.42
13	244@O15-K100@HZ1	9.94	2.80	151.00
14	244@O8-N188@HD21	2.46	2.83	156.80
15	244@O14-K100@HZ3	7.74	2.79	148.44
16	244@O15-K100@HZ2	9.31	2.80	152.45
17	244@O15-K100@HZ3	8.74	2.79	151.17
18	244@O14-N17@HD21	6.10	2.82	146.39

(b) the Y26-phospho system				
#	H-bond	Occupancy (%)	Distance (Å)	Angle (°)
1	244@O15-S23@HG	98.56	2.58	165.89
2	244@O8-Y92@HD21	12.51	2.65	166.63
3	244@O13-N17@HD21	85.67	2.75	147.20
4	244@O14-N17@HD21	77.63	2.89	145.58
5	244@O7-Y92@HH	7.18	2.67	162.81
6	244@O14-S23@HG	6.31	2.60	165.97
7	244@O13-K100@HZ3	7.74	2.73	155.89
8	244@O13-K100@HZ2	8.06	2.72	156.53
9	244@O8-N188@HD21	5.90	2.84	155.37
10	244@O9-K100@HZ1	5.89	2.76	157.02
11	244@O7-N188@HD21	5.86	2.84	150.15
12	244@O13-K100@HZ1	8.37	2.73	155.90
13	244@O9-K100@HZ2	11.10	2.76	153.49
14	244@O9-K100@HZ3	11.25	2.76	157.19
15	244@O13-S23@HG	3.08	2.60	166.03
16	244@O15-K100@HZ3	5.61	2.78	158.72
17	244@O15-K100@HZ2	5.23	2.77	157.81
18	244@O11-K100@HZ3	2.41	2.82	142.57
19	244@O11-K100@HZ1	2.26	2.83	140.79
20	244@O15-K100@HZ1	5.51	2.78	159.17
21	244@O11-K100@HZ2	1.99	2.82	142.97
22	244@O14-K100@HZ2	4.40	2.77	159.63
23	244@O14-K100@HZ1	5.12	2.77	159.91
24	244@O15-N17@HD21	77.32	2.69	145.96
25	244@O11-N17@HD22	3.76	2.79	153.11
26	244@O14-K100@HZ3	2.67	2.77	159.92
27	244@O7-N209@HD21	1.69	2.81	157.85
28	244@O8-K100@HZ1	5.52	2.76	155.11
29	244@O8-K100@HZ2	5.08	2.76	155.12
30	244@O8-K100@HZ3	4.95	2.76	155.14
31	244@O7-K100@HZ1	4.17	2.76	154.74
32	244@O7-K100@HZ2	3.80	2.77	153.48
33	244@O7-K100@HZ3	3.73	2.76	154.29

**Figure 4:**
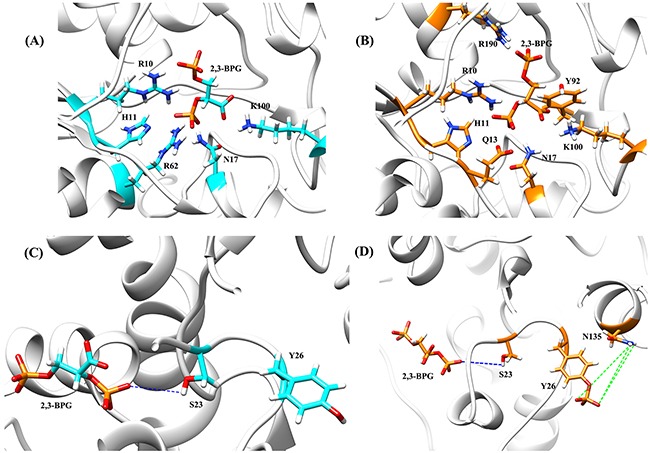
Structures of 2,3-BPG:PGAM1 complex for both the wild type system A and C. and the Y26-phospho system B and D 2,3-BPG, Y26, and their surrounding crucial residues are in stick representation. (A and B) 2,3-BPG is surrounded by a couple of ionic residues. (C) In the wild type system, the desired hydrogen bond 244@O15-S23@HG (the dashed blue line) is not formed due to the bad orientation between 2,3-BPG and S23. (D) In the Y26-phospho system, hydrogen bond 244@O15-S23@HG (the dashed blue line) is well formed, due to the three hydrogen bonds formed between the phosphorylated Y26 and N135 (the dashed green lines).

The hydrogen bond between 2,3-BPG and Serine 23 (S23) (presented as 244@O15-S23@HG in Table 1) is worth noting. It had a 98.56% occupancy in the Y26-phospho system, but was completely absent in the wild type system. The striking difference implies that Y26 played a critical role in stabilizing 244@O15-S23@HG. Given that Y26 is spatially close to S23, this is quite possible. Indeed, Y26 formed three hydrogen bonds with N135 (Table 2 and Figure 4D). These hydrogen bonds might keep S23 in an orientation that is suitable for forming 244@O15-S23@HG. The three hydrogen bonds were absent in the wild type system (Figure 4C).

**Table 2:** Properties of the hydrogen bonds formed between Y26 and its adjacent residues

(a) the wild type system			
H-bond	Occupancy (%)	Distance (Å)	Angle (°)
(b) the Y26-phospho system			
H-bond	Occupancy (%)	Distance (Å)	Angle (°)
None	N/A	N/A	N/A
Y26@O3P-N135@HD22	15.64	2.75	155.43
Y26@O3P-N135@HD21	8.66	2.85	160.23
Y26@O2P-N135@HD22	7.27	2.78	164.56

Besides S23, other residues such as Lysine 100 (K100) and Asparagine 17 (N17) also formed multiple stable hydrogen bonds with 2,3-BPG in the Y26-phospho system (Table 1(b)). A previous experimental study had also revealed the importance of K100 in 2,3-BPG binding [27]. Although K100 and N17 also mediated hydrogen bond formation in the wild type system, these hydrogen bonds had much smaller occupancy and were thus unstable (Table 1(a)). These data can explain the above RMSF analysis, which demonstrated that some residues (e.g., L18, E102, A105, D148) became less flexible upon Y26 phosphorylation (Figure 2B). For example, L18 is a neighbor of N17; thus hydrogen bonds formed by N17 would fix L18 as well, making the RMSF value at L18 greatly reduced. Similarly, the reduced flexibility of E102 and A105 might be due to the hydrogen bond formed by K100.

### Identification of an α-helix crucial for 2,3-BPG binding

To obtain a comprehensive understanding of structural variances of 2,3-BPG:PGAM1, snapshots of the trajectory data were clustered by using the average linkage algorithm; and five clusters were yielded respectively for each of the two systems. We then picked five representative structures *S_i_* (*i* = 0, 1, 2, 3, 4) from the five clusters, respectively. Note that *S*_0_ was fixed to be the initial structure. For the wild type system, we found that *S*_0_ was the largest cluster, containing 48.7% of the total snapshots. The structure *S*_0_ *wt* deviated from the crystal structure with a RMSD value 1.25*Å*. For the Y26 phospho system, we found that *S_1_* was the largest cluster, containing 89.2% of the total snapshots. The structure *S_1_* *phos* deviated from the crystal structure with a RMSD value 0.65 *Å*.

**Figure 5:**
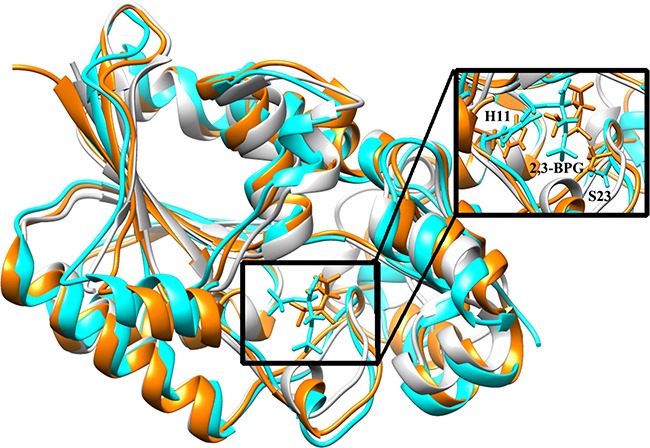
Superposition of *S_3_* *wt* (cyan), *S_0_* *phos* (orange), and the crystal structure 3FDZ (light gray) 2,3-BPG and some crucial residues of PGAM1 are in stick representation. The binding region is amplified.

**Figure 6:**
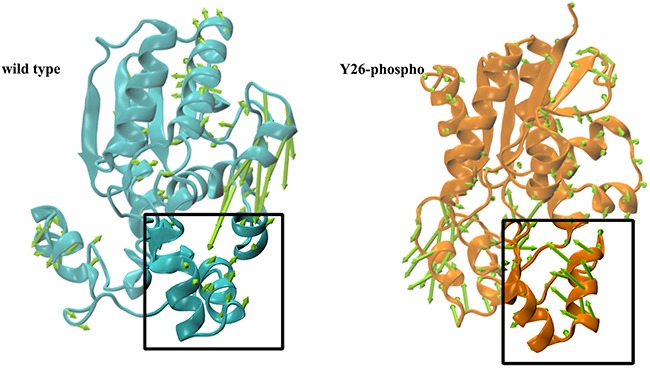
Porcupine plots for *S_3_* *wt* (cyan) and *S_0_* *phos* (orange) The conic arrows indicate directions of movement corresponding to the first principal component. The α-helix region is boxed.

We then used the crystal structure (PDB ID: 3FDZ) as the reference to study the structural deviations of *S_0_* *wt* and *S_1_* *phos*, because the crystal structure had recorded faithfully the actual binding of 2,3-BPG with PGAM1. The three systems were first superimposed (Figure 5). As expected, the overall structures of the three systems were quite similar. The area around the 2,3-BPG binding site showed relatively great variances, including an *α*-helix formed by residues Asparagine 99 to Glycine 108 (Figure 6). The α-helix was of particular importance, because one of its constituents, K100, had been shown important for the binding of 2,3-BPG [27]. In accordance with that, we also found that K100 formed hydrogen bonds of high occupancy with 2,3-BPG in the Y26-phospho system but not in the wild type system (Table 1). The α-helix thus deserved a more detailed analysis.

We first measured the α-helix's center of mass for all the three structures. We then calculated the mass center deviation (relative to the crystal structure) for both *S_0_* *wt* and *S_1_* *phos*, which were 3.62 *Å* and 1.38 *Å*, respectively. This result indicated that the α-helix of the Y26-phospho system was less deviated from the actual binding position than the wild type system (Figure 5). More insights can be obtained if we study the motion of the whole system, particularly the α-helix region. To that end, we applied principal component analysis (PCA) to both systems. For each system, we identified two principal components, which together represent more than 50% of the overall movements. The direction of movement corresponding to the first principal component is indicated by the conic arrows in Figure 6, which shows vividly the radical difference between the two systems: the α-helix moves away from the binding site in the wild type system; while it moves towards the binding site in the Y26-phospho system. The latter movement might bring K100 to the proximity of 2,3-BPG, facilitate the formation of stable hydrogen bonds, and ultimately confer a tight binding of 2,3-BPG to PGAM1.

### 2,3-BPG binding was an energy favorable event in the Y26-phospho system but not in the wild type system

**Figure 7:**
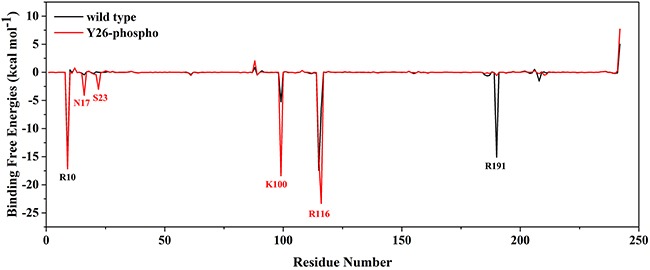
Per-residue binding free energies ΔGbindi (*i* = 2,3,…,243) of the wild type system (black) and the Y26-phospho system (red) Residues with highly negative binding energy values are labeled.

Affinity of enzyme-substrate binding can be estimated by calculating the free energy of binding Δ*G_bind_*, using methods such as MM/GBSA [13–15, 28]. We extracted 5000 snapshots from the last 200 ns trajectory and used the data to calculate Δ*G_bind_*. The results are presented in Table 3 and Supplementary Figure S3. The binding free energy for the Y26-phospho system was lower than the wild type system, which indicated that the 2,3-BPG molecule bound to the PGAM1 protein more tightly in the Y26-phospho system than in the wild type system. To identify residues that are crucial to the binding, we calculated per-residue binding free energies ΔGbindi(*i* = 2,3,…,243) (Figure 7). One sees that the wild type system had only one primary contributor Arginine 10 (R10); while the Y26-phospho system had three primary contributors: R10, K100, and Arginine 116 (R116). A residue far away from the binding pocket of 2,3-BPG, namely R191, was also worth noting. In the wild type system, R191 had much higher binding free energy than in the Y26-phospho system, which made it interact actively with 2,3-BPG and drag 2,3-BPG away from the binding pocket. This reduced the binding affinity of 2,3-BPG to the wild type PGAM1.

**Table 3:** Total binding free energy (kcal mol^−^^1^) and its components in the 2,3-BPG:PGAM1 complex for the wild type and Y26-phospho systems

System	ΔEele	ΔEvdw	ΔGGB	ΔGSASA	ΔGMM/BGSAa	−TΔS	ΔGbindb
wt	−324.78±80.02	−4.08±4.39	333.38±74.20	−3.32±0.28	1.21±9.96	18.60±5.07	19.81±11.18
Y26	−438.62±16.31	−2.70±3.44	441.46±12.73	−2.97±0.046	−2.82±5.25	15.08±2.42	13.25±5.78

^a^ Δ*G_MM/PBSA_* = Δ*E_ele_* + Δ*E_vdw_* + Δ*G_GB_* + Δ*G_SASA_*.

^b^ Δ*G_TOT_* = Δ*G_MM/GBSA_* − *T*Δ*S*.

To understand why the binding was easy and tight in the Y26-phospho system but not in the wild type system, we examined individual energy and entropy terms in the binding free energy expression (Table 3). One sees that for both systems, the primary force favorable to the binding was the electrostatic force Δ*E_ele_*: it was very negative (*−*324.78 kcal·mol^−1^) in the wild type system and was even more negative (*−*438.62 kcal·mol^−1^) in the Y26-phospho system. This is not strange, because 2,3-BPG is a highly negatively charged molecule, while its nearby PGAM1 residues are mostly positively charged residues such as K100 and R10, as revealed by the clustering analysis. This favorable electrostatic environment notwithstanding, the highly positive Δ*G_GB_* = 333.38 kcal·mol^−1^ made the binding unfavorable, in the wild type system. In the Y26-phospho system, electrostatic forces exerted by residue R10, K100, R116, S23, and N17 increased markedly (Figure 7), making the binding favorable.

The tightly bound 2,3-BPG might interact more actively with its surrounding residues, including K100 and R116, than in the wild type system. Note that K100 belongs to the α-helix discovered in the cluster and PCA analyses. Therefore, the active interaction might bring K100 closer to 2,3-BPG, and further attracted the α-helix moving towards 2,3-BPG (Figure 6).

### Y26 phosphorylation of PGAM1 enhanced glycolysis

PGAM1 catalyzes the eighth step of glycolysis, namely the isomerization 3PG → 2PG via the intermediate 2,3-BPG. Phosphorylation of PGAM1 at Y26 was hypothesized as an important mechanism to enhance glycolysis and consequently tumor growth [2].

**Figure 8:**
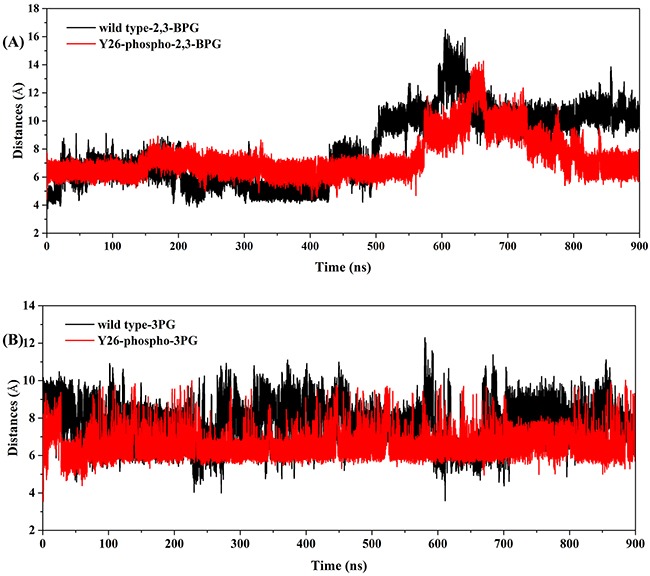
The distance plot over the 900 ns simulation time for both the wild type system (black lines) and the Y26-phospho system (red lines) **A**. The distance between P6 atom of 2,3-BPG and NE2 atom of H11. **B**. The distance between O atom around the C-2 position of 3PG and P atom of H11.

To test this hypothesis, we had studied the second half reaction of the isomerization: 2,3-BPG → 2PG. As mentioned above, these studies had been centered on the binding of 2,3-BPG to PGAM1, namely how Y26 phosphorylation stabilized the binding of 2,3-BPG to PGAM1. How about the subsequent events? Would Y26 phosphorylation help the production of 2PG, namely the transfer of the phosphoryl group from the C-3 position of 2,3-BPG to H11 residue of PGAM1? The superimposition in Figure 5 had suggested that the distance and orientation between the donor and receptors are better in the Y26-phospho system than in the wild type system. To test the intuition, we monitored the distance between P6 atom of 2,3-BPG and NE2 atom of H11 over the 900 ns simulation. We found that the distance fluctuated fiercely all over the simulation in the wild type system, with the largest distance even reaching ~16 Å (Figure 8A, black curve). In the Y26-phospho system, the fluctuation was much smaller (Figure 8A, red curve). At the end of the simulation, the distance in the Y26-phospho system was smaller than in the wild type system. These data suggested that the transfer of the phosphoryl group is much easier to occur in the Y26-phospho system, due to the overall closer distance between the donor and the receptor.

We then studied the effect of Y26 phosphorylation to the first half reaction of isomerization: 3PG → 2,3-BPG, also catalyzed by PGAM1. To evaluate the transfer of the phosphoryl group from H11 of PGAM1 to 3PG (whereby 2,3-BPG is obtained), we monitored the distance between P atom of H11 and O atom around the C-2 position of 3PG over the 900 ns simulation. We found that in the wild type system the distances fluctuated fiercely all over the simulation, with the highest value around ~11 Å (Figure 8B, black lines). In the Y26-phospho system, the fluctuation was much smaller (Figure 8B, red lines). The average values were 8.52 *Å* and 6.34 *Å* for the wild type and Y26-phospho systems, respectively. These data suggested that the phosphoryl group on H11 is much easier to transfer to the C-2 position of 3PG with the aid of Y26 phosphorylation.

Finally, we calculated the binding free energy of 3PG:PGAM1 (the reactant complex form) and 2PG:PGAM1 (the product complex form) and presented the results in Tables 4 and 5, respectively. For both 3PG:PGAM1 and 2PG:PGAM1, Y26 phosphorylation significantly reduced the binding free energy. Therefore, the function of Y26 phosphorylation is consistent throughout the entire eighth step of glycolysis. It stabilized the binding of all the three (3PG, 2,3-BPG, 2PG) to PGAM1 and thus secured the isomerization.

**Table 4:** Total binding free energy (kcal mol^−^^1^) and its components in the 3PG:PGAM1 complex for the wild type and Y26-phospho systems

System	ΔEele	ΔEvdw	ΔGGB	ΔGSASA	ΔGMM/BGSAa	−TΔS	ΔGbindb
wt	−129.63±36.76	−9.70±3.68	164.97±33.56	−3.26±0.17	22.39±6.44	16.60±5.00	38.99±8.15
Y26	−136.26±94.44	−7.88±3.24	161.95.46±40.20	−2.59±0.46	15.23±11.20	16.89±5.83	32.12±12.63

^a^ Δ*G_MM/PBSA_* = Δ*E_ele_* + Δ*E_vdw_* + Δ*G_GB_* + Δ*G_SASA_*.

^b^ Δ*G_TOT_* = Δ*G_MM/GBSA_* − *T*Δ*S*.

**Table 5:** Total binding free energy (kcal mol^−^^1^) and its components in the 2PG:PGAM1 complex for the wild type and Y26-phospho systems

System	ΔEele	ΔEvdw	ΔGGB	ΔGSASA	ΔGMM/BGSAa	−TΔS	ΔGbindb
wt	−241.54±51.55	−3.34±3.95	210.04±45.69	−3.37±0.14	−38.21±9.76	18.33±6.11	−19.88±11.51
Y26	−202.55±28.12	−3.00±4.06	156.75.46±25.36	−3.54±0.087	−52.35±9.20	16.89±5.83	−35.46±10.89

^a^ Δ*G_MM/PBSA_* = Δ*E_ele_* + Δ*E_vdw_* + Δ*G_GB_* + Δ*G_SASA_*.

^b^ Δ*G_TOT_* = Δ*G_MM/GBSA_* − *T*Δ*S*.

**Figure 9:**
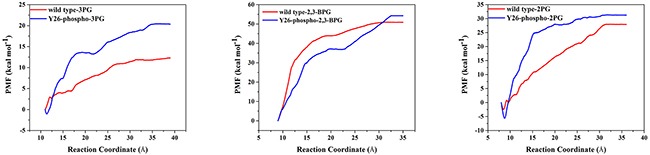
The PMF of pulling 3PG, 2,3-BPG, and 2PG from PGAM1 Red and blue lines are for the wild type and Y26-phospho systems, respectively.

We then performed ASMD [29] simulations to study unbinding of the three ligands (2PG, 2,3-BPG and 3PG) from PGAM1. The detailed description of ASMD can be found in the Method section. The results were presented in Figure 9. One sees that all the three ligands were harder to dissociate from the PGAM1 protein in the Y26-phospho system than in the wild type system. That is, the energy barriers of the Y26-phospho system were all significantly higher than the wild type system. These results were consistent with the MM/GBSA free energy calculations. All the ligands bound more tightly in the Y26-phospho system than in the wild type system.

The isomerization manifested an “induced fit” characteristic of binding. That is, the binding affinity became increasingly greater as the substrate changed from 3PG to 2,3-BPG and then to 2PG, because Δ*G_bind_*_:3*PG*_ > Δ*G_bind_*_:2,3−*BPG*_ > Δ*G_bind_*_:2*PG*_ (Tables 4 and 5). The order was followed by both the wild type and Y26-phospho systems.

## CONCLUSION

As a crucial pathway of cellular metabolism, glycolysis is almost always accelerated in cancer cells to produce a vast amount of energy to sustain their rapid growth and division. Glycolytic enzymes are therefore often upregulated in cancer cells, including PGAM1, the enzyme catalyzing the eighth step of glycolysis. Hitosugi *et al*. hypothesized that the phosphorylation of Y26 residue of PGAM1 greatly enhances PGAM1 activity and contributes to carcinogenesis.

To test the hypothesis, we constructed two *in silico* PGAM1 systems with Y26 either dephosphorylated (the wild type system) or phosphorylated (the Y26-phospho system). We then compared the two systems in terms of structural stability, hydrogen bond formation, binding free energy, etc, by MD simulations. We found that Y26 phosphorylation enhances the binding of PGAM1 to its substrates through generating electrostatic environment and structural features that are advantageous to the binding. For example, several Y26-phospho mediated hydrogen bonds facilitate the suitable orientation and distance between PGAM1 and its substrate. We also identified an α-helix whose location and movement are both suitable for the substrate binding in the Y26-phospho system but not in the wild type system.

Our studies have revealed considerable atomistic details of PGAM1, its substrates, and their interactions, which may provide valuable insights into computer-aided design of drugs that specifically target cancer cells. Through virtual screening of chemical libraries, for example, small drug molecules may be found that can greatly weaken or even block the binding between PGAM1 *phos* and its substrates, while having little effects on PGAM1 *wt*.

## materials and METHODS

### In silico models

PGAM1 is a dimer of two identical monomers. According to the crystal structure 4GPZ (resolution: 1.65 Å), the monomer consists of 242 amino acids (Alanine 2 to Methionine 243). We constructed an *in silico* model of the monomer by taking atomic coordinates from the crystal structure. This model was called PGAM1 *wt*. Because Y26 phosphorylation was the focus of the present study, we phosphorylated Y26 with Discovery Studio 4.0 Visualizer and created a new model PGAM1 *phos*. The protonation states of both models were carefully inspected based on the H++ on-line server [30], with the results presented in Supplementary Table S1. According to the server calculation, all the histidine residues were set to HIE.

Our PGAM1 models were then docked with 2,3-BPG. Because H11 in the unbound (apo) PGAM1 should be dephosphorylated but it was phosphorylated in the 4GPZ crystal structure, we used Discovery Studio 4.0 Visualizer to dephosphorylate H11 before the docking. The atomic coordinates of 2,3-BPG were obtained from the crystal structure 3FDZ [31], which was a 2,3-BPG:PGAM1 complex obtained from bacterium *burkholderia pseudomallei*. To replace bacterial PGAM1 with human PGAM1, we used the “superimposition method” [14, 32], which is effective in generating new protein/ligand complexes from old ones. Supplementary Figure S2 showed that 4GPZ and 3FDZ had high sequence identity (58.1%) especially for the ligand binding residues. We first superimposed our PGAM1 model on 3FDZ and achieved a 0.80 Å root-mean-square-deviation (RMSD) of superimposition. By removing all the PGAM1 atoms of 3FDZ, the desired human 2,3-BPG:PGAM1 complexes were created. The part of the complex centering around 2,3-BPG is shown for both the wild type system (Figure 4A) and the Y26-phospho system (Figure 4B). For both systems, 2,3-BPG is surrounded by a couple of ionic residues, which conforms with previous results that 3PG binds to an ionic pocket of PGAM1 [33]. This implies that the obtained 2,3-BPG:PGAM1 models are reasonable for MD simulations.

### Molecular dynamics simulation

All the MD simulations were performed by using AMBER14 package [34] with ff14SB force field [35]. The force field parameters for the phosphorylated Y26 and 2,3-BPG were provided by Homeyer's phosphotyrosine parameter set [36] and the general AMBER force field (GAFF) [37], respectively. All the missing hydrogen atoms of PGAM1 were added by the LEaP module. Sodium ions were added by using coulomb potential grid to neutralize the whole system. The two systems were then respectively solvated in an octahedral periodic box, with TIP3P water model [38]. The distance between the outermost protein atoms and the walls of the simulation box was set to be 8.0 Å. With 8 Na^+^ and 8116 water molecules added, the wild type system had 28251 atoms in total. With 10 Na^+^ and 8271 water molecules added, the Y26-phospho system had 28721 atoms in total. The following procedures were applied to each of the two systems.

- **Minimization** Energy Minimization was performed to obtain a low-energy starting conformation for the subsequent MD simulations. A total of 10000 steps of Minimization were performed: 4000 of steepest descent followed by 6000 of conjugate gradient. The whole system (including the protein, the ligand, Na^+^, and the water molecules) was first minimized, followed by minimization on the protein and ligand only.
- **Heating** The system was then heated under NVT conditions (canonical ensemble) from 0 to 300 K for 300 ps, with the Langevin thermostat applied. The force constant for the harmonic restraint was set to be 10.0 kcal mol^−1^ Å^−2^.
- **Equilibration** The system was then equilibrated for 10 ns under NPT conditions (with constant pressure 1.0 bar). The relaxation time for barostat bath was set to be 2.0 ps.
- **Simulation** The system was finally simulated for 900 ns under NPT and periodic boundary conditions, with the time step set to be 2 fs. The long range electrostatics was handled by the particle-mesh Ewald (PME) method [39]. The cut-off value for short range interactions was set to be 10.0 Å. Bonds involving hydrogen atoms were constrained with SHAKE algorithm.

### Trajectory analysis

A MD simulation usually generates a large bulk of data in the form of motion trajectories of all the atoms in the system. Valuable information can by yielded by analyzing the trajectories. In the present study, trajectories were analyzed with AmberTools 15.

- **Hydrogen bond** formation is sensitive to structural changes of biomolecules. In the present study, we used the following two criteria to judge whether or not a hydrogen bond is formed between an acceptor heavy atom A, a donor hydrogen atom H, and a donor heavy atom D. First, the distance between A and H is less than the distance cutoff 2.9 Å. Second, the A-H-D angle is greater than the angle cutoff 120°.
- **Clustering**. In the present study, a 900 ns MD simulation yielded 450000 snapshots (frames) of the trajectory. These snapshots were clustered into several groups, each containing molecular structures that are similar to each other. We used an average-linkage algorithm for clustering [40]. Each snapshot started as its own cluster; and the two closest clusters (judged by their distance) were merged in each iteration. The algorithm halted if the desired number *n* (here *n* = 5) of clusters had been obtained. The distance between clusters A and B was defined as the average of all the distances between a and b, where a (b) is a snapshot in the cluster A (B). We picked five representative structures *S_i_* (*i* = 0, 1, …, 4) from the five clusters, respectively. Note that *S*_0_ was fixed to be the initial structure.
- **Principal component analysis** (PCA) can be used to distinguish few dominant motions of the system (the principal components) from many fluctuations that are of little functional importance [41]. In the present study, PCA was rendered by ProDy [42]; structural visualization and analysis were rendered by software VMD [43] and Chimera [44].

### Binding free energy calculations

The free energy of receptor-ligand binding was calculated by the MM/GBSA method [13–15, 28]. To identify the most crucial residues of PGAM1 for the binding of 2,3-BPG, the total binding free energy was decomposed into contributions from individual residues (*i* = 2, 3, …, 243):

$$\Delta G_{bind}=\sum_{i=2}^{243}G_{bind}^{i}=\sum_{i=2}^{243}\sum_{j\neq i}^{243}\Delta G_{bind}^{i,j}$$

where ΔGbindi are the per-residue contributions, ΔGbindi,j are the residue-pairwise interaction contributions. The calculations were rendered by the MMPBSA.py module [45] of AMBER14.

### Adaptive steered molecular dynamics

Adaptive Steered Molecular Dynamics (ASMD) has been proved to be a powerful tool in studying dissociation of small ligands from proteins [29]. It was thus used to investigate the unbinding pathway of ligands from PGAM1. For an ASMD calculation, the overall reaction coordinate is divided into several stages. During each stage, we performed 40 Steered Molecular Dynamics (SMD) simulations following the approach described in [46], with the speed of the pulling force set to 1Å/ns. At the end of the stage, 40 trajectories had been generated, from which the potential of mean force (PMF) was calculated according to Jarzynski's equality [47]. The work done by the pulling force along each trajectory was calculated as well. To select the initial structure of the next stage from the ending conformations of all the 40 trajectories, we followed Hernandez and coworkers [29] by selecting the one whose corresponding trajectory had the work value closest to the PMF value. For determining the reaction coordinate, we applied tunnel analysis (see *Supporting Information* for details) implemented with the software package CAVER 3.0 and presented the results in Supplementary Figure S1. The reaction coordinate was determined to be the center of mass distance between the ligands (2PG, 2,3-BPG and 3PG) and Glu89. The initial coordinates of the ligands (2PG, 2,3-BPG and 3PG) were selected from the representative structures of cluster analysis of the last 200ns of the total 900ns conventional MD simulation. At each stage of the ASMD simulation, 40 SMD simulation were performed, with the pulling speed set to 1Å/ns.

## SUPPLEMENTARY MATERIALS FIGURES AND TABLES


