# Supplementary material for: Molecular dynamics simulation reveals how phosphorylation of tyrosine 26 of phosphoglycerate mutase 1 upregulates glycolysis and promotes tumor growth
Source: Oncotarget. 2017 Jan 5;8(7):12093–107. doi: 10.18632/oncotarget.14517 (PMC5355328; doi:10.18632/oncotarget.14517)
Supplement: Supplementary file 1 [file oncotarget-08-12093-s001.pdf]

Molecular dynamics simulation reveals how phosphorylation of tyrosine 26 of phosphoglycerate mutase 1 upregulates glycolysis and promotes tumor growth

SUPPLEMENTARY DATA

Tunnel analysis

Tunnel analysis was carried out by using the CAVER 3.0 software package [1]. CAVER is a software tool widely used for identification and visualization of tunnels and channels of a specified protein structure. Based on the trajectory generated by MD simulations, CAVER 3.0 was capable of finding the possible egress tunnels from the binding side to the surface of the enzyme, enabling automatic analysis of large ensembles of protein conformations. Several pathways with nearly identical axes may be classified into one cluster. The lowest cost pathway is selected and all the other pathways in the cluster are discarded. A trajectory of MD simulation serves as the input, while the detailed characteristics of individual transport pathways and their time evolution are the outputs. The mass centers of His11, Glu89 and Arg90 were chosen as the starting point for tunnel searching. For each trajectory, 1000 snapshots were extracted from the last 100 ns simulation. The probe radius and the clustering threshold were set to 1.0 and 8.5, respectively. For the other parameters, their default values were taken throughout the calculations. The obtained tunnels were visualized with VMD (Supplementary Figure 1).

Generation of 2pg and 3pg complexes

The crystal structure 3FDZ [3] recorded the accurate atomic coordinates of both bacterial PGAM1 and 2,3-BPG. The crystal structure 4GPZ) [2] recorded only human PGAM1. To create human PGAM1 in complex with 2,3-BPG, one has to dock the 2,3-PBG molecule into the active site of 4GPZ. Here, the superimposition method was used to obtain the complex structure. The sequence identity between 3FDZ [3] and 4GPZ was 58.1% and the result of sequence alignment was shown in Supplementary Figure 2.

Then the two crystal structures were superimposed. After the superimposition, the 3FDZ protein structure was removed, leaving a complex formed by 2,3-BPG and 4GPZ, namely the desired human 2,3-BPG:PGAM1 complex. The 3PG:PGAM1 complex was obtained by the superposition of chain B of 3FDZ and chain A of 4GPZ (The 3FDZ crystal structure contained two chains: chain A was in complex with 2,3-BPG and chain B was in complex with 3PG). In contrast, the 2,3-BPG:PGAM1 complex was obtained by the superposition of chain A of 3FDZ and chain A of 4GPZ. In the protein data bank, we did not find the crystal structure of 2PG:PGAM1. Therefore, the 2PG molecule should be docked into the active site of PGAM1 in order to create the 2PG:PGAM1 complex structure. Here, AutoDock4.2 software package [4] was employed for the docking. The parameter set for docking calculations is summarized as follow:

The 2PG molecule was docked into the active site of PGAM1 employing the Auto Dock tools (ADT). The nonpolar hydrogen atoms were removed and only the polar hydrogens were kept. The Gasteiger charges were added to the PGAM1 and 2PG molecules. A box size of 40×40×40 Å^3 with grid spacing 0.375 Å was defined around the binding site of PGAM1 so that it contained all the residues that are critical for interacting with 2PG. Here, Arg10, His11, Ser23 and Lys100 were selected as the active site residues interacting with 2PG. The grid map around the binding site of PGAM1 was generated by the probe atoms employing the Auto Grid program. Each grid in the map represents the potential energy of a probe atom in the presence of all the atoms of the receptor molecule. The Lamarkian Genetic Algorithm (LGA) was used for docking study. One hundred runs with 15000000 maximum evaluations and 270000 generations were used for docking simulation. The docking pose with the lowest binding energy −4.47 kcal mol^{-1} was chosen as the starting structure for the subsequent MD simulation.

To obtain reasonable structures before generating force field parameters, the three ligands 3PG, 2,3-BPG, and 2PG were optimized by employing Gaussian 09 software package [5] using DFT method (B3LYP) [6] under 6-31G basis set. The force field parameters for these three ligands were generated by Antechamber module in AMBER14 [7].

Binding free energy calculations

Based on the last 200ns simulation of each system, binding free energies were calculated by using MM/GBSA method. For each system, 160 frames were extracted from the last 200ns trajectory files. For all the 160 frames of each system, the binding free energy calculations all took the entropy into consideration. The entropy was calculated by using Normal Mode Analysis (nmode). The results are presented in Supplementary Figure 3.

As Supplementary Figure 3 shows, the binding free energies of all the wild type systems went through larger fluctuations than Y26-phospho systems. The binding free energies of Y26-phospho systems were quite stable during the last 200ns simulation. Besides, the binding free energies of Y26-phospho systems were all lower than those of wild type systems, which implied that all the three molecules (3PG, 2,3-BPG and 2PG) bound more tightly with PGAM1 *phos* than with PGAM1*wt*.

REFERENCES

1. Petřek M, Otyepka M, Banáš P, Košinová P, Koča J and Damborský J. CAVER: a new tool to explore routes from protein clefts, pockets and cavities. BMC Bioinformatics. 2006; 7:1.
2. Hitosugi T, Zhou L, Fan J, Elf S, Zhang L, Xie J, Wang Y, Gu T-L, Alečković M and LeRoy G. Tyr26 phosphorylation of PGAM1 provides a metabolic advantage to tumours by stabilizing the active conformation. Nat Commun. 2013; 4:1790.
3. Davies DR, Staker BL, Abendroth JA, Edwards TE, Hartley R, Leonard J, Kim H, Rychel AL, Hewitt SN and Myler PJ. An ensemble of structures of Burkholderia pseudomallei 2, 3-bisphosphoglycerate-dependent phosphoglycerate mutase. Acta Cryst F. 2011; 67:1044-1050.
4. Morris GM, Huey R, Lindstrom W, Sanner MF, Belew RK, Goodsell DS and Olson AJ. AutoDock4 and AutoDockTools4: Automated docking with selective receptor flexibility. J Comput Chem. 2009; 30:2785-2791.
5. Frisch M, Trucks G, Schlegel H, Scuseria G, Robb M, Cheeseman J, Scalmani G, Barone V, Mennucci B and Petersson G. 09, Revision D. 01, Gaussian. Inc, Wallingford, CT. 2009.
6. Becke AD. Density functional thermochemistry. III. The role of exact exchange. J Chem Phys 1993; 98:5648-5652.
7. Case D, Babin V, Berryman J, Betz R, Cai Q, Cerutti D, Cheatham Iii T, Darden T, Duke R and Gohlke H. Amber 14. 2014.

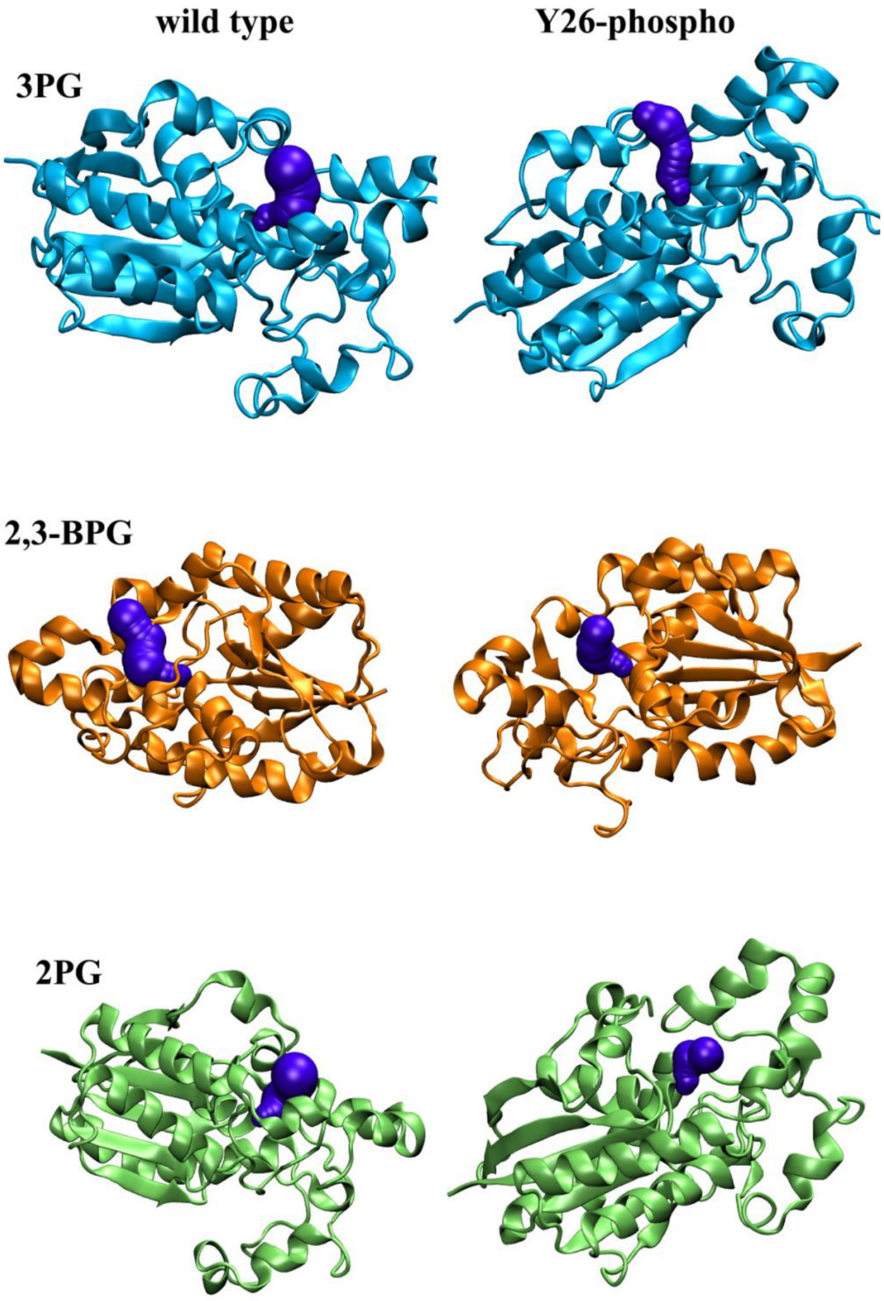

Supplementary Figure 1: The highest possible tunnels (colored in purple) for 3PG, 2,3-BPG, and 2PG egressing from PGAM1. The direction of the pathway was chosen as the pulling direction in the ASMD simulations.

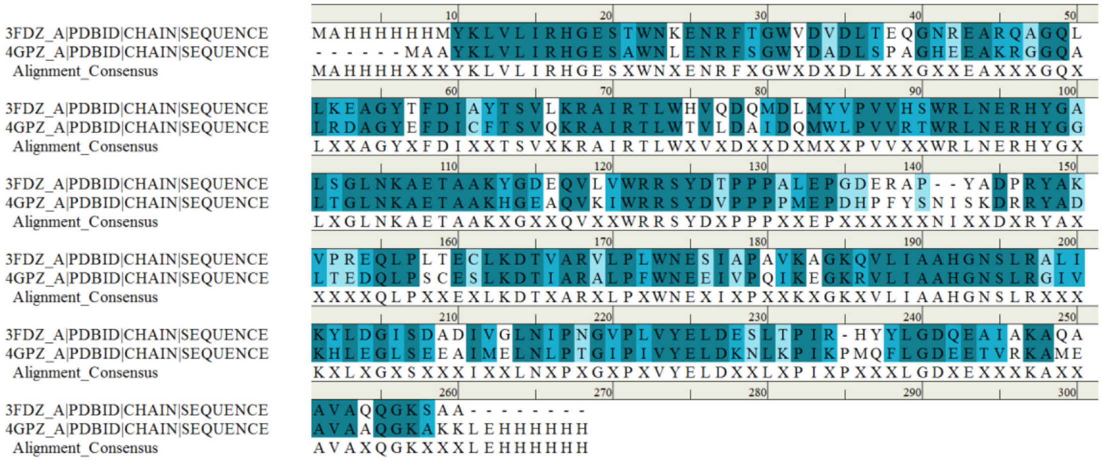

Supplementary Figure 2: Sequence alignment between 4GPZ and 3FDZ obtained with Discovery Studio 4.0 Visualizer.

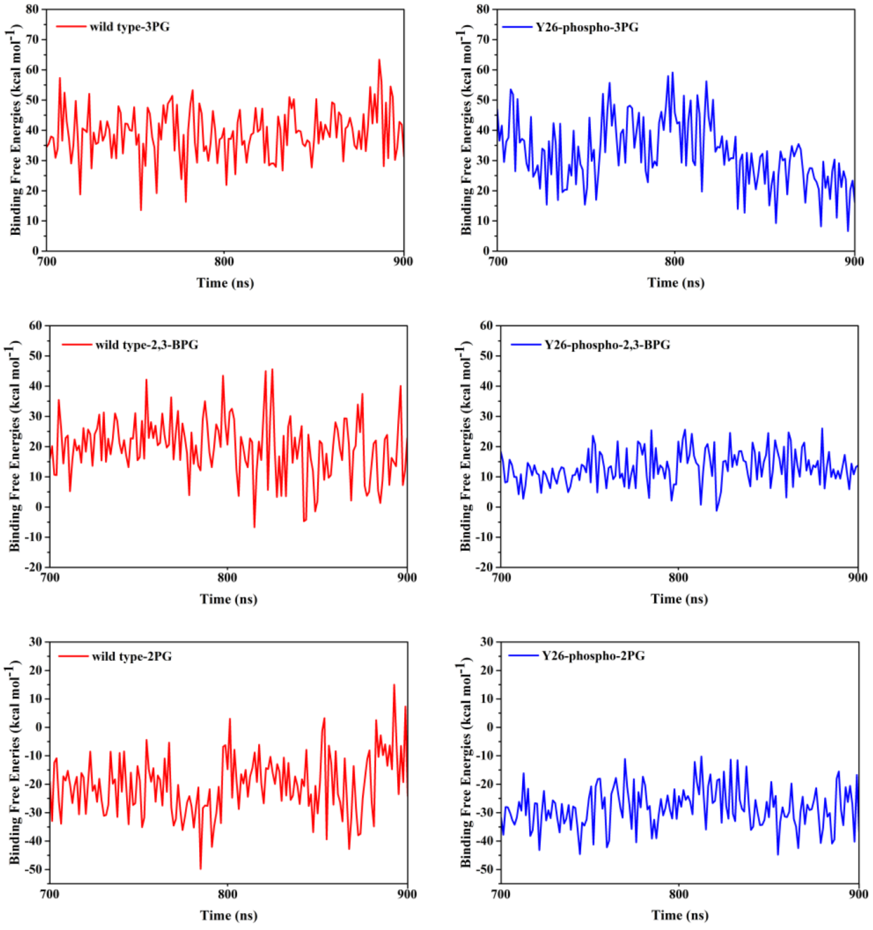

Supplementary Figure 3: Binding free energy (with entropy included) variations during the last 200ns for the binding of 3PG, 2,3-BPG, and 2PG to PGAM1.

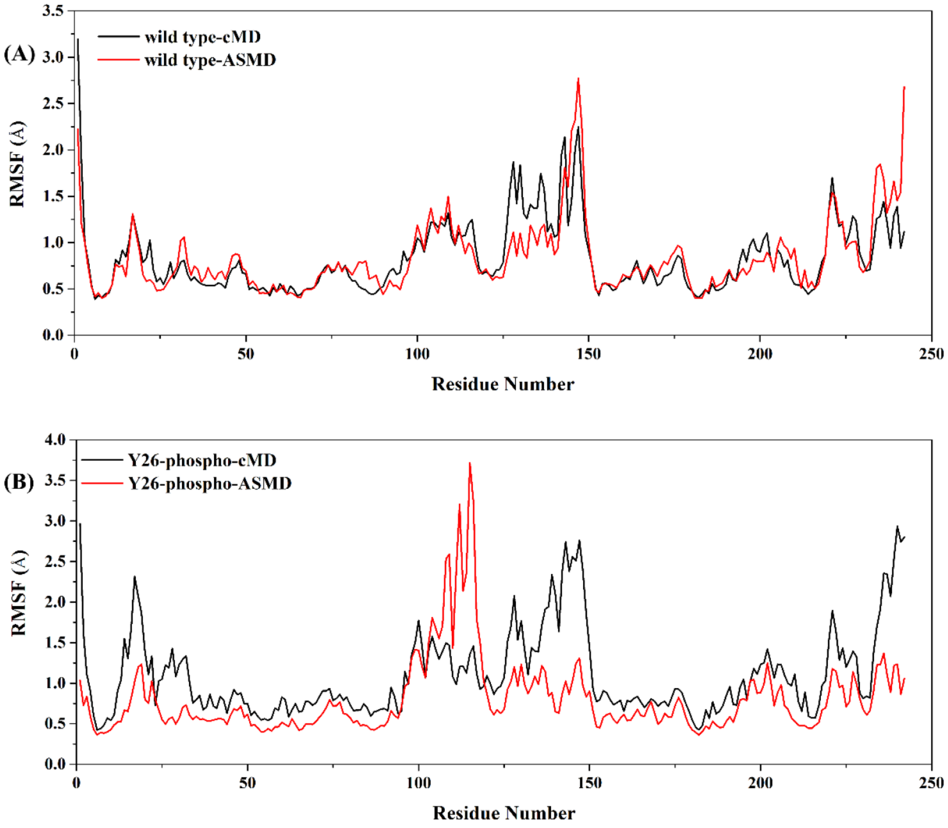

Supplementary Figure 4: The RMSF for the wild type and Y26-phospho 2,3-BPG:PGAM1. A. The wild type system RMSF obtained by cMD and ASMD simulations. **B.** The Y26-phospho system RMSF obtained by cMD and ASMD simulations.

Supplementary Table 1: The results of the H++ on-line server calculation for 2,3-BPG system

Histidine	PKa value
10	<0.0
34	5.8
90	<0.0
106	6.2
129	4.0
195	4.8
